# Effect of Laparoscopic Sleeve Gastrectomy on Hypothyroidism in Patients with Morbid Obesity: A one-year follow-up Prospective Cohort Study

**DOI:** 10.30699/ijp.2024.2026732.3290

**Published:** 2025-01-10

**Authors:** Nasser Malekpour Alamdari, Iman Ansari, Mohammad Ali Azizi Nadian, Mahdiyeh Sadat Seyyedi, Maryam Abbasi

**Affiliations:** 1 *Critical Care Quality Improvement Research Center, Shahid Modarres Hospital, Shahid Beheshti University of Medical Sciences, Tehran, Iran *; 2 *General Surgery Department, Shahid Modarres Hospital, Shahid Beheshti University of Medical Sciences, Tehran, Iran*

**Keywords:** Bariatric surgery, Hypothyroidism, Morbid obesity, Sleeve gastrectomy

## Abstract

**Background & Objective:**

Obesity is a growing public health problem worldwide. Bariatric surgery is considered the safest and most effective therapeutic option, although it is associated with various metabolic and neurohormonal consequences. This study aims to evaluate the effect of sleeve gastrectomy (SG) on hypothyroidism within one year following surgery.

**Methods:**

In this single-center prospective cross-sectional study, patients with morbid obesity who were candidates for SG were included. Body mass index (BMI), levothyroxine dosage, and thyroid function tests were compared at baseline and 12 months after surgery.

**Results & Conclusion:**

Patients’ BMI, levothyroxine dosage, TSH, and T4 levels decreased significantly after surgery (*P*<0.0001). The effect of SG on T3 levels was not statistically significant. The results of this study demonstrate that SG can significantly improve thyroid function tests in patients with morbid obesity and reduce their need for levothyroxine within one year following surgery.

## Introduction

Obesity is an increasing global concern that negatively impacts individual health. Its prevalence has doubled over the last 40 years, and more than 600 million adults and 100 million children are obese (1). Obesity is associated with numerous complications, such as type 2 diabetes, cardiovascular disease, and certain cancers, and it increases the risk of all-cause mortality (2). Although lifestyle changes are the mainstay of obesity management, many patients fail to achieve effective and durable weight loss (3). Morbid obesity is defined as a body mass index (BMI) greater than 40 in a healthy individual or greater than 35 in someone with an underlying condition such as cardiovascular disease or diabetes, and it typically requires therapeutic intervention. For these patients, bariatric surgery (BS) is a widely accepted and increasingly performed safe and effective method with long-lasting results (4).

Bariatric surgery promotes weight loss primarily by reducing food intake, interfering with nutrient absorption, and altering neurohormonal signaling (5,6). Its effects include reductions in leptin, ghrelin, appetite, and systemic inflammation, as well as improvements in insulin resistance and glucose metabolism (7,8). However, the metabolic effects of BS extend beyond adipose tissue–derived hormones and metabolism (9–11).

Hypothyroidism is the most prevalent hormonal disorder associated with obesity. There is a close relationship between obesity and thyroid function; it has been suggested that, similar to insulin resistance and type 2 diabetes, obesity can lead to resistance to thyroid hormones, resulting in hypothyroidism. Conversely, hypothyroidism can promote metabolic diseases such as obesity by affecting dietary intake and energy expenditure (12). Growing evidence supports changes in thyroid hormone levels following BS, although studies on the effects of BS on thyroid dysfunction have produced controversial results (13–15). This study aims to investigate the changes in thyroid hormones one year after laparoscopic sleeve gastrectomy (SG) in obese patients with hypothyroidism who were referred to a tertiary hospital in Tehran, Iran.

## Materials and methods

### Study Design

This prospective cross-sectional study was conducted prospectively with a one-year follow-up based on the STROCCS statement in 2024 at Modarres Medical Training Center, Tehran, Iran (16). 

### Subjects

Patients with a body mass index (BMI) >40 kg/m² who were candidates for SG and had a documented history of overt hypothyroidism (abnormal thyroid stimulating hormone [TSH] levels or chronic use of levothyroxine) were eligible to enroll in the study. Patients with concomitant renal or hepatic failure, cardiovascular diseases (such as triple-vessel disease, advanced heart failure, or a history of myocardial infarction), psychological problems (such as schizophrenia, bipolar mood disorder, or major depression), those who did not consent to participate, or those who failed to complete the follow-up period were excluded. Additionally, patients with morbid obesity secondary to any genetic or hormonal disorder were excluded.

All included patients underwent a thorough history taking and physical examination. Levothyroxine dosage, BMI, serum TSH, thyroxine (T4), and triiodothyronine (T3) levels were recorded at baseline and 12 months after surgery. It should be noted that all patients were operated on by a single laparoscopic surgeon, and all tests were performed at both time points in the same laboratory using the same kit.

### Statistical Analysis

Data were analyzed by statistical package for social sciences (IBM Corp. Released 2019. IBM SPSS Statistics for Windows, Version 26.0. Armonk, NY: IBM Corp). The quantitative and qualitative ordinal variables were expressed as mean ± standard deviation (SD) and percentage, respectively Also, paired sample t-test, Chi-square, and correlation tests were used to assess the post-operative changes of variables and determine associated factors. The significance level was considered P-value<0.05.

## Results & Discussion

A total number of 60 patients completed the one-year follow-up. Thirty-six patients (60%) were female, and 24 patients (40%) were male. The mean age of patients was 29.85 ± 7.14 (ranging from 17 to 44) years. The mean weight and BMI before surgery among the patients were 114.90±13.86 kg and 43.47±2.51 kg/m^2^, respectively. Also, the mean levels of TSH, T4, and T3 before and after the surgery are shown in Table 1. While the Levothyroxine pill taken, BMI, TSH, and T4 decreased significantly 12 months after surgery (*P*<0.001), the T3 levels remained similar to the baseline (*P*=0.42) (Table 1). 

**Fig. 1:**
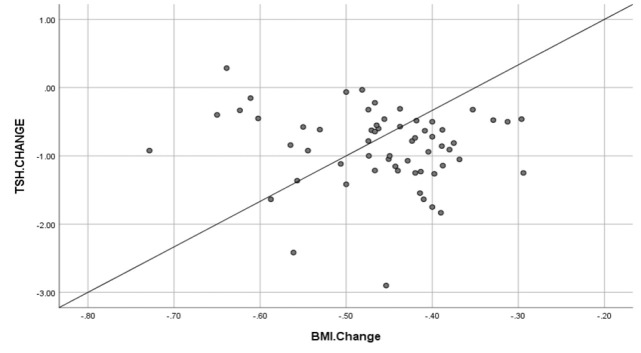
The correlation between initial BMI and final thyroid stimulating hormone (TSH)

The correlation between BMI and final TSH was examined using Pearson's correlation coefficient, which was not significant (*P*=0.1, r= 0.35) (Figure 1).

Today's world, with its changing lifestyle and technological advancements, which are associated with inactivity and improper nutrition, has provided a basis for the increasing prevalence of obesity. Obesity is a global problem that has raised both mortality and morbidity. It is associated with 120 million disability-adjusted life-years, and more than 2 trillion dollars are spent annually on obesity-related medical costs (1). Obesity has been proven to be linked with metabolic diseases. It is still not entirely clear whether obesity leads to thyroid dysfunction or if hypothyroidism contributes to obesity. However, it can be stated that hypothyroidism is the most common metabolic disorder in patients with obesity. Among the various treatment methods for obesity, bariatric surgery has gained great popularity as the most definitive option (12,13). It has been suggested that bariatric surgery is effective in treating hypothyroidism in obese patients by improving metabolic pathways, though opinions differ (14,15). Therefore, this study was conducted with the aim of prospectively investigating the effect of sleeve gastrectomy (SG) on thyroid hormone levels in obese patients with hypothyroidism.

The results of this study showed that during one year of follow-up after SG, patients’ weight and BMI significantly decreased. Thyroid hormone levels also decreased significantly during this period compared to pre-surgery levels, although the effect of SG on T3 was not statistically significant. Similar to our findings, several studies, such as those by Azran* et al.* (13), Khan* et al.* (17), and Juiz-Valiña* et al.* (18), reported a significant reduction in TSH levels and a decrease in levothyroxine dosage. However, some studies, such as those by Najjari* et al.* and Karaman* et al.*, reported that T4 does not change significantly after bariatric surgery (14,19).

It is noteworthy that the beneficial effects of bariatric surgery on thyroid hormones are not limited to overt disorders; bariatric surgery, particularly SG, can also improve subclinical hypothyroidism (20,21). Yska* et al.* also investigated the long-term dynamics of thyroid hormones over four years following surgery. Their study showed that during the first two years post-surgery, thyroid hormone levels frequently fluctuated and required regular monitoring to adjust medication accordingly. However, the hormone levels stabilized thereafter, and less frequent monitoring proved sufficient (22). Another study by Julià* et al.* revealed differences between SG and Roux-en-Y gastric bypass surgery regarding levothyroxine dosage two years after surgery—the SG group required significantly lower dosages (23). The hypothalamic-pituitary-adipose (HPA) axis theory may explain the underlying mechanism for this interaction. In patients with obesity, there is a central resistance to TSH, and weight loss following bariatric surgery ameliorates this resistance, similar to the mechanisms that reduce insulin resistance and facilitate insulin activity (15,25). Leptin, a key hormone produced by adipose tissue and involved in HPA signaling, may decrease TSH levels following Roux-en-Y gastric bypass (26).

The factors associated with thyroid hormone dynamics are not yet fully understood. Similar to our study, other studies have not found a significant correlation between BMI and TSH (27). Additionally, bariatric surgery techniques appear to correlate with the final thyroid function; according to Dewantoro* et al.*, euthyroidism or a reduction in levothyroxine occurs more in patients who undergo restrictive procedures compared to those who undergo combined restrictive/malabsorptive techniques (28).

Although our study has provided important insights into the effects of SG on thyroid hormones, it has several limitations: it was conducted at a single center, had a relatively small sample size, and assessed only SG. We highly recommend that further research be designed as multicentric studies with extended follow-up periods, comparing the results among different bariatric surgery techniques.

## Conclusion

The results of this study demonstrated that SG could significantly improve the thyroid function tests in patients with morbid obesity and decrease the patient’s need for Levothyroxine within the one year following surgery.

**Table 1:** The initial and final states of measured variables after one-year follow-up.

Variables	Unit	The initial value (mean± SD)	The final value (mean± SD)	P-value
**Weight**	Kg	114.90±13.86	85.70±9.04	0.00
**BMI**	Kg/m^2^	43.47 ± 2.51	29.85 ± 1.95	0.00
**Levothyroxine dosage**	Number of 100 µg pills taken	0.22 ± 1.00	0.42± 0.24	*P*<0.0001
**TSH**	µIU/mL	0.84±3.52	0.7±2.02	*P*<0.0001
**T4**	µg /dL	1.20±5.46	0.83±5.00	0.01
**T3**	ng/mL	0.21±0.79	0.17±0.76	0.42

SD: Standard deviation; BMI: Body mass Index; TSH: thyroid stimulating hormone; T4: Thyroxine; T3: Triiodothyronine
